# Some Developments in Abdominal Surgery

**Published:** 1918

**Authors:** F. Lace

**Affiliations:** Honorary Surgeon, Royal United Hospital, Bath


The Bristol

Medico-Chirurgical Journal.

*" Scire est nescire, nisi id me*
*Scire alius sciret."*

SPRING NUMBER, 1918.

### SOME DEVELOPMENTS IN ABDOMINAL SURGERY.

The Presidential Address, delivered on 12th December, 1917, at the opening of the Forty-fourth Session of the Bristol Medico-Chirurgical Society.

BY

F. LACE, F.R.C.S.,

*Honorary Surgeon, Royal United Hospital, Bath.*

In taking the Presidential Chair of this Society, I feel that I must again express my very great appreciation of the honour you have done me in electing me to this position, and all the more so in that I think this is only the second occasion upon which a member of the Society non-resident in Bristol or Clifton has been nominated as President. I shall always consider it the greatest compliment which my professional friends in this city could have paid me.

When I think of the distinguished men, many of whom were my teachers thirty years ago, who have occupied this chair, it seems almost presumptuous to attempt to follow in their footsteps. However, I will do my best, and being fairly well conversant with the affairs of the Society, hope that I may carry out my duties to your satisfaction.

The foremost and most difficult of those duties is that of addressing you on this occasion. Two great difficulties beset me. In the first place, in these times of stress it is more than difficult to get through one's daily work, much more to find sufficient time to give adequate thought to the subject of an address to a scientific society. In the second place, the fact of not being connected with a School of Medicine, as practically all my predecessors have been, is a real handicap. One misses the incentive to take a comprehensive and detailed grasp of the subject in view, and the facility of language which must come to anyone daily occupied in imparting the knowledge which he has acquired.

I could have wished to address you on what I may call some by-path in our profession, such as that of the surgical superstitions which are still handed down to the student in our text-books. But time has not sufficed. Many other subjects have passed through my mind, but been abandoned for the same reason, and eventually my thoughts carried me back to my student days at the Royal Infirmary, and to one of my teachers whose name will always take a foremost place in the annals of this Society. I refer to Mr. Greig Smith. I was his dresser for an unusually long period, and it was impossible to be associated with him without being affected by his unvarying kindness, high ideals—as shown in his Presidential address to this Society—and enthusiasm in his work. To him must be ascribed my interest in the subject of Abdominal Surgery, and it is not too much to say that any success I may have achieved in practice is directly due to him more than to anyone else.

It was at this time, just thirty years ago, that he published the first edition of his *Abdominal Surgery*, a book which, as has been said, " filled a void much felt at the time of its first publication, and was welcomed in the warmest terms, not only in this country and America, but also on the continent." Upon taking down my copy and glancing through its pages once more, it occurred to me that it might not be amiss, after this lapse of time, to spend the few moments at my disposal this evening in considering some of the advances which have been made in this branch of surgery since this book was published.

The subject of Abdominal Surgery has, of course, become too vast to allow of any detailed review, but it may be useful to point out the general principles upon which such advances have been founded, and which in many instances do not seem likely to be improved upon.

And here may I recall the warning uttered by Greig Smith in his preface. He says: "From a practical point of view, it is expedient that the surgeon, who makes an abdominal section for a preconceived purpose, should be ready off-hand to deal with an unexpected contingency. The recondite nature of many abdominal diseases frequently necessitates conclusions to operative procedures very different from what were contemplated. Confidence and capacity on the part of the surgeon who ventures upon abdominal work can scarcely exist apart from a complete knowledge of all abdominal operations." I fancy that this warning is just as necessary nowadays, if not more so, as then. It is difficult enough for those who are almost daily opening the abdomen, to keep abreast of the many advances which are being constantly made in technique, etc., and yet many others are bold enough to undertake such work with the scantiest knowledge of the difficulties which they may have to face. How complicated even an operation for appendicitis may prove to be!

As in connection with all other branches of surgery one particular advance has taken place, the substitution of aseptic for antiseptic methods. In my student days the use of the carbolic spray was universal. All instruments, towels, sponges, and hands were soaked in the same fluid, much to the detriment of one's skin, and one left the operating theatre saturated inside and out with the antiseptic. How much simpler and more secure is sterilisation by steam and boiling, and how much irritation of the tissues, both of the patient and operator, is avoided. I mentioned sponges just now. Swabs and pads of various sizes and material have taken the place of marine sponges, but I sometimes wonder whether we were wise in abolishing the large, flat sponges, quite easily sterilised, as Lister pointed out. Has anything been devised quite so efficient for the purpose for which it was used, the "packing-off" of the site of an operation in the abdomen ?

As regards diagnostic methods, there is little to add to the very complete chapters on the subject in the work which serves as my text-book. The discovery of the X-rays had not then been made. To-day we could not well do without this method of examination in conjunction with the bismuth meal. The diagnosis of pathological conditions of the stomach by these means has been materially improved, particularly in America ; and how useful it is to be able to sort out cases of real mechanical obstruction of the bowels from amongst the myriads of people suffering from that popular condition styled "intestinal stasis." How many less exploratory operations for renal calculus are now performed, since the presence of a stone in any part of the urinary tract can be almost certainly demonstrated to us on a photographic plate.

Speaking of the kidney reminds me of another means of diagnosis not in use in those days, but which has now for a long time become a routine practice. I refer to the cystoscope, with or without the ureteral catheter. This instrument now enables us to diagnose with precision conditions formerly characterised by a considerable degree of doubt, and in some instances to apply local treatment.

I will now consider some of the more common abdominal operations seriatim, and will commence with :—

*Ovariotomy.*—This may be looked upon as the pioneer operation of abdominal surgery. By the time Greig Smith's book was written the operation had reached a stage of finality which has not been improved upon since. The extra-peritoneal treatment of the pedicle by use of a clamp had been abolished, as had also the treatment by clamp and cautery, vicissitudes through which other procedures have passed, and the simple intra-peritoneal method of ligature had been generally adopted. All the difficulties and complications which might arise in the course of the operation were well recognised and described, and the practice of the operation remains to-day where it was then.

But when we come to the consideration of the removal of the uterine appendages there is more to be said. I do not refer to the removal of the ovaries and Fallopian tubes for inflammatory conditions, but to oöphorectomy performed for the purpose of producing an artificial menopause, for functional neuroses, hystero-epilepsy, menorrhagia, or for the cure of uterine fibro-myoma. Greig Smith fully discussed the arguments for and against Batty's operation, as it was called, and considered it of value in certain conditions, even going so far as to say that in the treatment of hystero-epilepsy the results were promising, and we used to see very many more ovaries removed in those days than now. I believe and hope that oöphorectomy for such conditions is rarely adopted nowadays. In fact, the wholesale removal of ovaries seemed at one time to be a source of discredit to the profession.

The removal of the appendages, with a view to preventing the growth and causing the atrophy of uterine myomata, falls under a different category, and appeared in those days to be likely to be generally adopted, and it was foretold that it would in the future mean fewer hysterectomies. This prophecy, however, has not held good, and I suppose the operation is now never performed for this purpose, mainly because we soon learnt that such tumours do not of necessity undergo atrophy after the menopause, to say nothing of the dangerous degenerative changes which not infrequently occur in them.

*Operations upon the Uterus.*—In the first place I take *Hysterectomy*, which in 1887 was rarely performed for any condition other than malignant disease or fibro-myoma. For malignant disease the operation was performed by the vaginal route. Greig Smith said that the abdominal operation was "almost universally discarded," and that with a proper selection of cases "recurrence is almost certainly not more rapid than in other operations for cancer, and permanent recovery is just as likely to be secured." In many cases partial operations were performed, and when the disease appeared to be confined to the cervix, it was considered that amputation of this portion alone was called for.

I have never performed the operation of vaginal hysterectomy, for the results of the cases which I saw in my early days were not encouraging; but there is no doubt that it was a very satisfactory operation in good hands, and the mortality in those days was decidedly lower than in the high operation. As time went on we realised that, as in other regions, the whole secret of success in the removal of malignant disease lay in the wide excision of all possibly infected tissues, particularly of lymphatic vessels and glands, and this could not be satisfactorily accomplished in the case of the uterus per vaginam. And so we came to adopt the operation by the abdominal route, now spoken of as the Wertheim operation. By this technique we are enabled to remove the parametric tissues as widely as possible, and also the lymphatic glands in the pelvis and iliac regions, or higher if necessary. The risk of wounding the bladder and ureters, so frequent an accident in vaginal hysterectomy, is thus minimised—though apparently it does still occur—and it is possible to resect them if necessary. The scope of the operation has also been widened, many a case which appears too far advanced for removal from below proving amenable to operation from above. Unfortunately the proportion of cases that we come across that are not too advanced for removal is still very small, and one almost despairs of any improvement in this respect.

All the hysterectomies for fibro-myoma that I saw in those days—and they were few in number—were performed by the use of Koeberle's wire clamp, with extra-peritoneal treatment of the stump. The broad ligaments were usually included in the stump, but Greig Smith recommended that they should be ligatured and divided in some difficult cases in order to obtain a more satisfactory pedicle. Intra-peritoneal treatment of the pedicle did not give satisfactory results, the mortality being given as 56.2 per cent., as against 33.3 per cent. with the extra-peritoneal method. Greig Smith did, however, write that "no doubt the advancing surgery of the day will not rest until some intra-peritoneal method has been devised which will excel all others." Such a method was somewhat slow in evolution, and it has always seemed surprising to me that we did not earlier take to deliberate ligature of the broad ligaments and uterine arteries, whereby the total or sub-total removal of a fibro-myomatous uterus has become in many cases an almost bloodless operation, and the mortality has been reduced to insignificant proportions. As in so many other operations, the advance has been entirely towards simplicity of technique.

Of *Cæsarean Section* I will say but a few words. The operation as described thirty years ago is that of to-day. The results are better owing to the fuller recognition of the fact that early operation is the chief factor making for success. The operation is much more frequently performed. Indeed it is a question whether it is not being to some extent abused. Taking cases at all stages of labour, the mortality is still by no means negligible, and what is a comparatively easy operation should not serve as an excuse for obstetrical ignorance.

There is a class of operations on the uterus, not practised in my early days, which deserves mention, namely the various procedures which have been adopted for the treatment of displacements, more particularly procidentia. The varieties of method recommended are innumerable, ranging from a simple ventro-fixation, or plication of the round ligaments, to splitting of the organ and incorporation of the segments in the abdominal wall, and their very number only goes to prove that none is of anything like universal benefit. Nevertheless, I have long been of opinion that it were better if practitioners would advise their patients to undergo some simple surgical treatment in the early stages of this condition, rather than to condemn them to the prolonged use of that objectionable instrument, the pessary.

*Operations on the Stomach.—Gastrostomy* was a well-recognised procedure. Greig Smith considered that the operation was "systematically delayed too long," a fault much less applicable nowadays. But he was "of opinion that considerable weight ought to be given to the desires of the patient after an honest and impartial statement of the possible and probable results have been put before him. My experience is that he elects not to be operated upon." The operation which he elaborated and described was excellent in those days, when the chief aim was to obtain firm adhesion of the stomach to the abdominal wall before opening it.

The improvement in technique which soon followed consisted in the addition of a channel through which the tube is passed, such channel being provided by infolding the stomach walls by one or other of several methods. Thus the tube can be introduced at once, food immediately given, and leaking prevented. Such is the operation of to-day, and one feels far more justified in recommending it, the immediate result and prognosis being so much improved, especially if not too long delayed. It is surely worth while to be spared death from starvation.

Operation for *Perforating Gastric Ulcer*, although described, I never saw whilst at the Royal Infirmary, and I believe it was some years later when it came into general use. Presumably this was because the condition was not diagnosed in a sufficiently early stage, as no doubt perforation of these ulcers was as frequent then as now. Nowadays there is seldom any difficulty in the diagnosis, and a large proportion of the cases are saved, either by simple suture, or suture in conjunction with gastro-enterostomy. Whether or not the abdomen should be washed out in these cases is still a vexed question. I have recently read an account of a series of cases in which lavage was not once used, and in which it is condemned; but in all my cases—a considerable number—I have invariably practised copious irrigation, and have had no reason to regret it.

For *Pyloric Obstruction* dilatation or pyloroplasty were the operations usually performed. Anterior gastro-enterostomy was described as a method of treatment, but had seldom been employed. The posterior operation had not then been practised, and even ten years later Greig Smith wrote that the results were such that the operation had been abandoned, kinking of the jejunum at the site of anastomosis being the objection. This method, however, was soon after generally adopted, and the danger of kinking avoided by making the point of junction as near the commencement of the jejunum as possible, instead of at a point about a foot lower down, as was at first recommended. I must confess that I never could understand why the method of leaving a loop was ever adopted, except perhaps that it made the necessary manipulations easier, and after my first operation I abandoned it for the more common-sense no-loop method, though I had not seen it described. In these days there is a considerable variety of operations in use for the purpose of producing an anastomosis between the stomach and duodenum or jejunum, which I need not go into. They are all useful under the varying circumstances which may be met.

There has been much difference of opinion as to whether this short-circuiting operation should be recommended in cases of malignant disease of the stomach. My experience is that if taken in time it usually gives the greatest relief, prolonging life sometimes for upwards of a year, and at any rate preventing death from starvation.

*Pylorectomy* or partial *Gastrectomy* had seldom been performed, and had given very fatal results. Gradually the technique of these operations has improved, so that the operative mortality has been reduced to reasonable proportions. Also by an accurate knowledge of the lymphatic system of the stomach we have learnt how much of the organ and which of the neighbouring lymphatic glands should be removed, in order to reduce the chances of recurrence of the disease as far as possible. Probably the most recent method of completing a gastrectomy by uniting a long loop of the small intestine directly to the divided stomach will prove to be a decided advance, the length of the operation being thereby considerably shortened.

Before leaving the subject of operations upon the stomach, may I express the opinion that the greatest desideratum always has been, and still is, some means of early and accurate diagnosis of the conditions, which can only be effectively treated surgically if recognised in an early stage. The greatest advance appears to me to have been made by the use of the bismuth meal and X-ray examination, and I have been particularly struck by the painstaking and excellent result which has been accomplished by these means in the United States.

Next I come to the subject of operations for the relief of *Intestinal Obstruction.* Greig Smith gave us in his work much and most excellent advice on this subject, advice which has been reiterated by every writer since. In those early days practically no cases were handed over to the surgeon until treatment by drugs had been prolonged to the utmost limit, and it was necessary to state, as he did, that "if we are doubtful whether we can do good by operating, we may be certain that there are many ways of doing harm by administering drugs." These criticisms have had their effect, and large though the mortality still is from acute intestinal obstruction, it cannot be now said that there is much undue delay in seeking surgical interference.

As regards operative details, it was then, probably for the first time, urged that "no operation for intestinal obstruction is properly completed if the patient leaves the table with a greatly distended abdomen, evacuation of the contents being an essential proceeding." With this end in view, Greig Smith advised a temporary enterostomy in cases in too serious a condition to admit of any prolonged operation. I wonder how many lives have been saved by this simple procedure.

Short-circuiting by means of a lateral or other form of anastomosis, as a temporary or permanent measure of relief for obstruction, was a later development, and, as you are aware, is now frequently practised.

In the treatment of Intussusception we do not now waste valuable time in efforts at reduction by insufflation, but open the abdomen and reduce it manually, thus greatly reducing the mortality.

Another operation which I do not recollect seeing when a dresser was *Resection of the Intestine.* A good many cases had been recorded, and it was then regarded as one of the most successful of heroic operations. The mortality was mainly due to peritonitis resulting from some defect in the operation, and no doubt the better results of the present day are largely due to improved methods of preventing contamination of the peritoneal cavity, *i.e.* the careful "packing-off" of the operation area. Although there is still a large variety of modes of suture described, probably in practice but few are in general use. Nor is there any call for any but the simplest methods, such as will fulfil two or three essential requirements. Generally speaking, continuous sutures have taken the place of interrupted, and taking the usual plan of two layers of suture, the inner one should aim at inverting the cut edges of the gut and bringing the serous surfaces into apposition, at the same time controlling hemorrhage; whilst the outer should be such as, when tightened, is as far as possible buried, so minimising the risk of subsequent adhesions to neighbouring parts.

During the present war, when multiple wounds of the intestine have to be dealt with as rapidly as possible, it has been proved that a single line of suture penetrating the whole wall of the bowel is usually quite efficient. Mechanical aids, in the shape of cylinders and bobbins, have been discarded, the only exception being in a low resection of the rectum, where a large rubber tube, fixed into the proximal segment, is of the greatest assistance in invaginating the cut edges and allowing of efficient suturing in a difficult position.

The prognosis as regards recurrence after resection of malignant growths of the intestines has been much improved, in the same way as in connection with gastrectomy, namely by a thorough study of the lymphatic areas of the different portions of the bowel, and consequent wider removal. There seems to be no limit as to the amount of bowel which can be removed with impunity as far as nutrition is concerned. We are led to believe that the large intestine is of no use to us, if not a positive danger, and that we should live longer without it; and I can personally vouch for the fact that satisfactory nutrition can be obtained when all but about eighteen inches of the small intestine has been put out of action.

The question of operating for localised or general peritonitis is much too extensive to be dealt with now, but I may mention that I never saw the appendix vermiformis removed whilst I was at the Royal Infirmary, although it was well recognised as a cause of suppurative peritonitis. One can hardly realise the change which has taken place in practice in connection with this small portion of our anatomy, and I cannot help wondering whether the pendulum has not swung too far in the other direction. Every discomfort in the right side of the abdomen seems to be traced to the appendix, and the public have heard so much about appendicitis, and have such a dread of it, that upon the least provocation they demand its removal. None of us will deny that early operation and removal of the organ have saved very many lives; but must we not at the same time admit that very many unnecessary appendicectomies are performed when the organ is said to be "quiescent," but in reality has nothing the matter with it, and never had.

The best method of performing *Colotomy*—or *Colostomy*, as I think it should be called—had not, at the time of which I am speaking, been settled. The lumbar or extra-peritoneal route had for a long time been in general favour, and even then was usually chosen, the mortality being said to be less than that of the inguinal operation. But the anterior or trans-peritoneal method was being steadily revived, and not many years later was generally adopted, though the procedure was somewhat modified. In the older operation a portion only of the circumference of the gut was sutured to the parietes, but when it was revived it was found better to pull out an entire loop of bowel and to fix it in position by acupuncture needles, or by a glass rod or other instrument passed beneath it. Later it was proved that one or more sutures passing through the parietes on either side and beneath the bowel was quite sufficient to keep it in position, and this procedure has not been improved upon?

Operations upon the *Gall-bladder* and *Bile Ducts* were few in number, and I believe that I acted as "dresser" to the first case of the kind upon which Greig Smith operated, a case of a single gall-stone impacted in the cystic duct. Of course most of these operations are performed for the removal of biliary calculi, and as we came to recognise more fully the baneful effects of temporary or permanent biliary obstruction, so the number of operations increased. The modes of attacking calculi in the various parts of the ducts have been elaborated, and, notwithstanding the extreme difficulties often met with, the results are wonderfully good. Apart from obstruction to the flow of bile into the digestive tract, we know that there is great danger from septic absorption in these cases, and therefore it is almost universally considered that drainage of the gall-bladder or ducts is an essential finish to all such operations. That it is not essential to drain the common duct after the removal of a stone from its lumen I know, for I have always closed the incision in the duct without drainage, and without any ill-effects to my knowledge. As regards the gall-bladder it is different, for it is so frequently at least chronically inflamed, and hence is the source of the septic absorption that we fear.

If the gall-bladder appears to be in a normal condition, I

am one of those who see no objection to immediate closure, so that I have often performed what is styled the "ideal" cholecystotomy, widely though it is even now condemned. Again, it appears to me that when the viscus is evidently diseased it is a sound procedure to excise it, notwithstanding the loss of drainage facilities, for the septic focus is in this way removed, and the necessity for drainage avoided. Cholecystectomy is, therefore, more widely practised than formerly, and I find that I more often than not complete operations for gall-stones in this way. The fact that malignant disease of the gall-bladder is almost always due to chronic irritation by gall-stones is another strong argument in favour of its removal, when it is evidently in a state of long-standing inflammation. The argument that its removal would make any subsequent operation upon the bile-passages more difficult and severe should not be given too much weight, for the necessity for such, though possible, is highly improbable.

Before leaving the region of the liver, let me refer to the surgical treatment of *Cirrhosis* of that organ, producing ascites. This has gradually become an established procedure. The operative mortality was at first high, but has been largely reduced by the proper selection of cases. As you know, the desired result has been obtained by promoting adhesions between the liver and the diaphragm, or of the omentum to the abdominal parietes, or by drainage of the peritoneal cavity into the subcutaneous tissues. Opinions differ as to whether the improvement is due to the relief of the portal circulation by provision of a collateral venous flow, or by the improvement of the blood supply of the liver. Anyhow, it is an operation which might well be more often undertaken.

Operations upon the *Kidney*, *Ureter* and *Bladder* are not always considered to belong to the domain of abdominal surgery, although it is not quite clear why. It is true that they are generally conducted extra-peritoneally, but by no means always so. As stated earlier, the main improvement in the surgery of these organs consists in methods of diagnosis, due to the routine use of the cystoscope and ureteral catheter. There is little excuse now for attacking them without knowing definitely what the condition is with which we have to deal. I think Greig Smith must be acknowledged to have taken a foremost place in resuscitating suprapubic cystotomy, if only for the removal of calculi. It was not long, however, before it was adopted for the removal of innocent and malignant growths, as well for the enucleation of the hypertrophied prostate gland. As regards prostatectomy, I am tempted to express the opinion that the ultimate result of this operation is not by any means always so satisfactory as is generally represented. It is those of us who have prolonged care of our own cases, or the after-treatment of patients leaving the hands of others a few weeks after the operation, who realise the troubles which may occur owing to delay in healing of the wound in the base of the bladder, and the cicatricial contraction of its neck.

There are two other organs to which a short allusion should be made, the *Pancreas* and the *Spleen*. In regard to the former, it is cheering to find records of an increasing number of successful operations for Acute Pancreatitis by means of incisions and drainage. And as regards Splenectomy, although it was performed some few times many years ago, in a casual way, for "simple hypertrophy," yet it is only during the last few years that it has come to be adopted as a recognised method of treating Splenic Anæmia and other blood conditions. In such conditions it seems to promise at least relief from symptoms and prolongation of life in a fair proportion of cases, and is not unlikely to become more generally adopted.

Here I will bring to a close this very superficial and somewhat scrappy survey of some of the developments in Abdominal Surgery which I have witnessed. I have found it interesting to " think back " for a while, and to realise that the advances in surgery during our time have been very real ; that many procedures of thirty years ago have not been improved upon ; that many others, then only tentatively undertaken, have been generally adopted ; whilst still others, then hardly dreamed of, have been permanently added to our resources.

